# Carbon assimilation profiles of mucoralean fungi show their metabolic versatility

**DOI:** 10.1038/s41598-019-48296-w

**Published:** 2019-08-14

**Authors:** Julia Pawłowska, Alicja Okrasińska, Kamil Kisło, Tamara Aleksandrzak-Piekarczyk, Katarzyna Szatraj, Somayeh Dolatabadi, Anna Muszewska

**Affiliations:** 1 Department of Molecular Phylogenetics and Evolution, Faculty of Biology, Biological and Chemical Research Centre, University of Warsaw, Zwirki i Wigury 101, 02-089 Warsaw, Poland; 2 Institute of Biochemistry and Biophysics, Polish Academy of Sciences, Pawinskiego 5A, 02-106 Warsaw, Poland; 3 Faculty of Engineering, Sabzevar University of New Technology, Sabzevar, Iran

**Keywords:** Evolutionary ecology, Fungal evolution, Fungal physiology

## Abstract

Most mucoralean fungi are common soil saprotrophs and were probably among the first land colonisers. Although Mucoromycotina representatives grow well on simple sugar media and are thought to be unable to assimilate more complex organic compounds, they are often isolated from plant substrates. The main goal of the study was to explore the effects of isolation origin and phylogenetic placement on the carbon assimilation capacities of a large group of saprotrophic Mucoromycotina representatives (i.e. Umbelopsidales and Mucorales). Fifty two strains representing different Mucoromycotina families and isolated from different substrates were tested for their capacity to grow on 99 different carbon sources using the Biolog phenotypic microarray system and agar plates containing selected biopolymers (i.e. cellulose, xylan, pectin, and starch) as a sole carbon source. Although our results did not reveal a correlation between phylogenetic distance and carbon assimilation capacities, we observed 20 significant differences in growth capacity on specific carbon sources between representatives of different families. Our results also suggest that isolation origin cannot be considered as a main predictor of the carbon assimilation capacities of a particular strain. We conclude that saprotrophic Mucoromycotina representatives are, contrary to common belief, metabolically versatile and able to use a wide variety of carbon sources.

## Introduction

Although plant tissues are the most common carbon source on the earth’s surface, their carbon is hardly accessible for heterotrophic organisms because it is mainly present in the form of complex polymers. The main components of plant cell walls (representing up to 70% of the biomass) are polysaccharides – cellulose, hemicellulose and pectin. Among them, cellulose is the most ubiquitous and may constitute up to 30% of the mass of primary plant walls and up to 98% of the mass of secondary plant cell walls^1^.

Fungi have evolved to possess a number of mechanisms that allow them to decompose organic plant debris^2^ and thus play a central role in carbon cycling as they are the most efficient degraders of plant biomass. A popular hypothesis that widespread coal deposition during the Carboniferous Period was caused by a temporal lag between the evolution of abundant lignin production in woody plants and the subsequent evolution of lignin-degrading fungi has been rejected by Nelsen *et al*.^3^. However, it is widely accepted that lignocellulolytic enzymes evolved relatively late, only within the ancestors of Agaricomycetes (Basidiomycota), ca. 350 Mya^4,5^. Nevertheless, fungi probably established mutualistic symbiosis with the first land plant lineages already in Ordovician (ca. 450 Mya) and these first land plants’ partners most likely belonged to Mucoromycota phylum^6^.

The phylum encompass zygospore-forming fungi that share a common mainly plant-related nutritional mode^7^. It comprises three subphyla: Glomeromycotina (obligatory endomycorrhizal fungi), Mortierellomycotina, and Mucoromycotina. Mucoromycotina is, according to current knowledge, the most diverse group, comprising three orders: Endogonales, Umbelopsidales and Mucorales. While Endogonales representatives are mainly obligatory plant symbionts^8^, the remaining two orders (Umbelopsidales and Mucorales) are mainly ubiquitous plant related soil saprotrophs (and there are only rare examples of opportunistic pathogens from immunocompromised patients^9–11^)

In general, fungi obtain nutrients by secreting hydrolytic enzymes into their surroundings and absorbing the digested compounds. They rely mainly on the presence of hydrolytic enzymes and transporter proteins which mediate the translocation of molecules across cell membranes^12^. Therefore, a profile of potential nutrition capacities defines the role of the fungus in its environment and thus it can be assumed that the fungal lifestyle is reflected by the repertoire of secreted enzymes and possessed transporters^13^.

Saprotrophic Mucoromycotina representatives are commonly called “sugar fungi” as they grow well on simple sugar media, but in contrast to ascomycetes and basidiomycetes they are thought to be unable to assimilate more complex organic compounds such as cellulose and lignin^14^. On the other hand, recent papers conclude that *Mucor* representatives are able to produce polymer-degrading enzymes, e.g., amylases, xylanases, steroid 11α-hydroxylases, phytases, proteases, and lipases^15,16^. Moreover, the existing knowledge of the physiological capacities of saprotrophic Mucoromycotina representatives is still limited to a few well-studied taxa (e.g. www.fung-growth.org)^17,18^. Nevertheless, an experiment using a large number of representative taxa and measuring their direct ability to grow on different carbon sources could unambiguously determine whether saprotrophic Mucoromycotina representatives are only “sugar fungi” or much more versatile.

The main goal of the study was to use carbon assimilation capacities to explore the effects of isolation origin and phylogenetic placement on enzyme production by a large group of saprotrophic Mucoromycotina representatives (i.e. Umbelopsidales and Mucorales). Carbon utilisation profiles were assessed using phenotypic microarrays and the screening of enzymatic capacities was performed on polymer containing agar plates. We used this information to verify the hypothesis that the carbon assimilation properties depend on strain isolation origin rather than on its phylogenetic placement.

## Results

### Usage of simple carbon substrates

**Figure 1:**
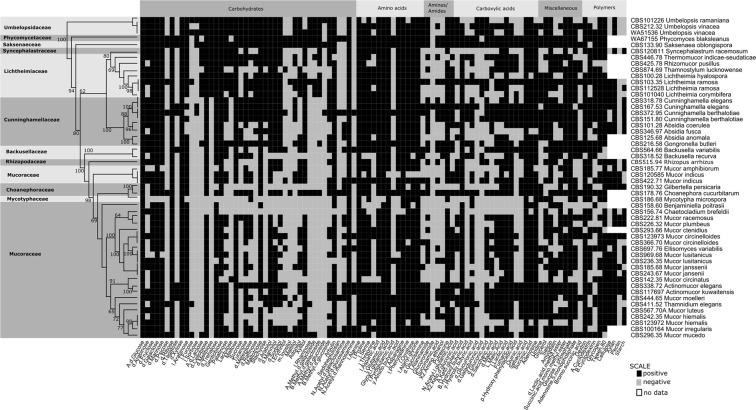
Heatmap representing carbon source utilisation capacity of 52 strains of saprotrophic Mucoromycotina obtained from Biolog FF MicroPlates assay and from experiment-determining growth capacity on selected biopolymers. RAxML phylogenetic tree is illustrating evolutionary relationships of tested fungal strains. Tested carbon sources are grouped into guilds according to Preston-Mafham *et al*.^36^.

**Figure 2:**
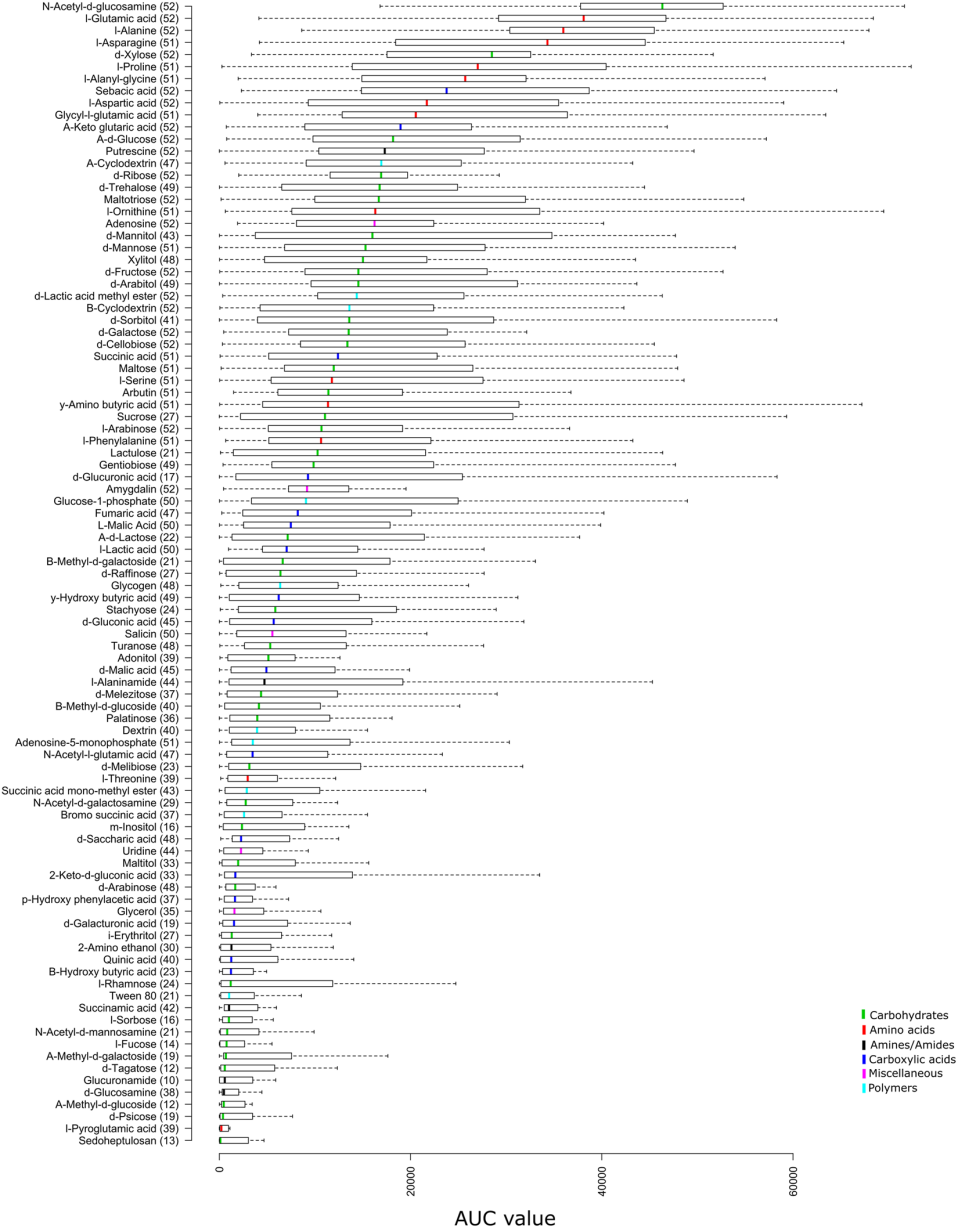
Carbon source utilisation by fungal strains represented as interquartile ranges of AUC values. Bar in the middle of the box represents the median and its colour indicates substrate type. Whiskers indicate the most extreme data point which is no more than 1.5 times the interquartile range from the box. Numbers in parentheses determine number of strains which were able to grow on a particular substrate.

The carbon assimilation profiles of 52 Mucorales strains were obtained by screening on Biolog microplates and are summarised in Fig. 1. None of the analysed strains were able to use all 95 carbon sources. On average, 60 substrates were absorbed per strain, i.e., approx. 63%. The number of substrates used by particular strains ranged from 27 (for *Benjaminiella poitrasii* and *Mycotypha microspora*) to 89 (for *Saksenaea oblongispora*). Only 6 carbon sources (α-D-glucose, D-ribose, D-xylose, L-alanine, sebacic acid and N-acetyl-D-glucosamine) were used by all strains and all of them grew fastest on N-acetyl-D-glucosamine. The capacity to use the remaining 89 substrates differed between the studied strains. Each of the 95 tested carbon sources was used by at least ten of the analysed strains. All of the studied fungal strains grew the fastest on amino acids which are known to constitute the main nutritional source for the majority of fungi^19^ (Fig. 2). Furthermore, monosaccharides were in general metabolised faster than more complex carbohydrates.

### Growth capacity on selected biopolymers

All 36 tested strains exhibited a capacity to grow on oat xylan as a sole carbon source, but only 25 of them were able to use cellulose (Fig. 1). The smallest number of strains (i.e. 10 out of 36) were able to grow on apple pectin as the only carbon source. It is noteworthy that all three representatives of Umbelopsidales were able to grow only on xylan. The capacities of the remaining strains to grow on starch, pectin and cellulose was strain dependent and was not correlated with isolation origin nor with phylogenetic placement.

### Factors shaping carbon usage capacities

**Figure 3:**
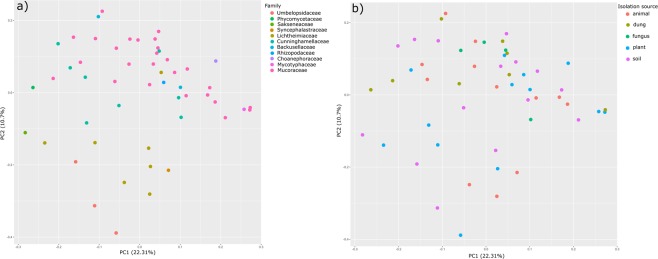
PCA of normalised mean AUC values derived from Biolog FF system for the 52 mucoralean strains. Colours indicate families (**a**) or substrate from which the strain was isolated (**b**).

Principal Component Analysis (Fig. 3) showed that phylogenetic placement (as family assignment) can explain the variability in the carbon assimilation profiles between the analysed fungi better than their isolation origin. The Kruskal–Wallis H test revealed significant differences (p < 0.05) in the usage of 6 carbon sources between groups of fungi isolated from different substrates and in the usage of 20 carbon sources between different family representatives (Supplementary Table S3).

**Figure 4:**
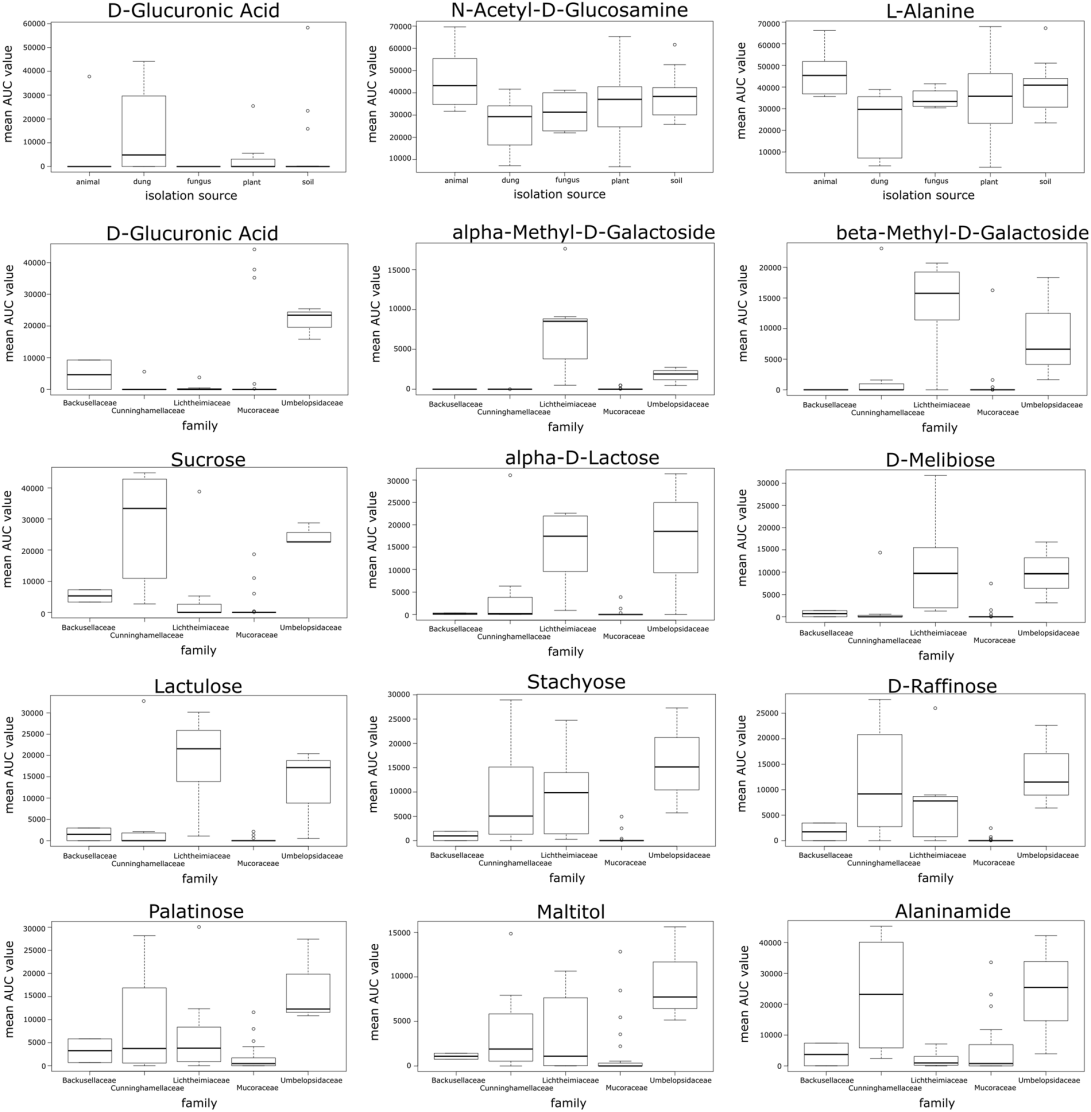
Utilisation of carbon sources for which the difference between groups was statistically significant in Kruskal-Wallis H test (p < 0.05). Grouping is done either by fungal families or substrate of origin. Utilisation is represented as interquartile ranges of AUC values with bar in the middle of the box representing median and whiskers indicating the most extreme data point which is no more than 1.5 times the interquartile range from the box.

Although the results of a Mantel test did not reveal a statistically significant correlation between phylogeny and carbon assimilation capacities (r = 0.15; p = 0.10), we identified carbon sources which are used differently by distinct phylogenetic groups (Fig. 4). The representatives of the Umbelopsidaceae family grew efficiently on several carbohydrates (α-D-lactose, D-melibiose, lactulose, α-methyl-D-galactoside, β-methyl-D-galactoside, sucrose, palatinose, stachyose, D-raffinose and maltitol), but were not able to grow on plant-related biopolymers such as cellulose, pectin or starch. Although the representatives of Lichtheimiaceae and Cunninghamellaceae used some of the abovementioned carbohydrates more efficiently than other families, there was no clear pattern differentiating the families. Some abilities to assimilate carbon sources were shared by Umbelopsidaceae and Lichtheimiaceae (e.g. α-D-lactose), others were characteristic of Umbelopsidaceae and Cunninghamellaceae (e.g. usage of alaninamide) whereas stachyose and D-raffinose were used by all three families (see Fig. 4). Interestingly, Mucoraceae representatives used carbohydrates less efficiently than fungi from other families but the majority of them (14 out of 18) were able to grow on cellulose as a sole carbon source.

### Transporter genes

The percentage of proteome which is occupied by transporter genes (from groups described in Schwartze *et al*.^20^: major facilitator superfamily – MFS_1: PF07690, MFS_1_like: PF12832, MFS_2: PF13347, ABC transporters – ABC_tran: PF00005, ABC2_membrane: PF01061, ABC_membrane: PF00664, PDR_CDR: PF06422, and other sugar transporters – Sugar_tr: PF00083) was compared between 19 available genomes of Mucorales representatives (see Supplementary Table S5 for genome data and transporter counts). This analysis revealed that the representatives of Lichtheimiaceae and Cunninghamellaceae family were characterised by a higher average number of sugar transporters and major facilitator superfamily 1 (MFS1) transporters in their membranes which may explain their more efficient growth on carbohydrates as the sole carbon source.

## Discussion

The main aim of this study was to explore the largely undescribed metabolic properties of Mucoromycotina. Since fungi display a great diversity of carbon assimilation capacities, we screened a collection of 72 strains of saprotrophic Mucoromycotina on Biolog phenotypic microarrays. Although this method enabled a rapid generation of large datasets, the repeatability of the results for some strains was low and led to the exclusion of some strains from the final analysis. The lack of efficient sporulation of some strains was probably the main reason for the high variability of the results, as previously reported by Baldrian *et al*.^21^. Another problem is that the most ubiquitous plant polysaccharides, such as cellulose and pectin, are not represented in Biolog FF microplates (https://biolog.com). Therefore, additional experiments on agar plates were also performed according to methods previously described by Farrow^22^ and Vermelho and Couri^23^. Combining information from Biolog FF microplates with additional experiments on plates made it possible to get sufficient data for further comparisons between carbon utilisation capacities of different fungal strains.

Fungi generally utilise carbohydrates through the production of carbohydrate hydrolytic enzymes secreted into the environment, which digest large organic molecules into smaller molecules which can then be absorbed as nutrients^24^. Several studies have already demonstrated a strong relationship between the repertoire of carbohydrate active enzymes (CAZymes, http://www.cazy.org^25^) in fungal genomes and their saprotrophic lifestyle^26–28^. However, this capacity is probably crucial mainly for organisms which degrade large organic molecules.

Saprotrophic Mucoromycotina representatives are commonly thought to be unable to assimilate more complex organic compounds such as cellulose and lignin^14^. For example, the analysis of the genome of *Rhizopus oryzae* revealed that it possesses pathways for the degradation of easily digestible plant cell wall saccharides, but it is unable to degrade cellulose^26^. In our study, several saprotrophic Mucoromycotina representatives, including *Rhizopus*, were able to grow on cellulose as a sole carbon source. This inconsistency could probably be explained by the fact that mucoralean cellulolytic enzymes can differ from their well-known Dikarya homologs and therefore are hard to detect in standard genome annotation procedures.

On the other hand, the cellulolytic activity was not detected in fungi from the Umbelopsidaceae family, which used simple carbohydrates more efficiently. However, fungi from this group are often isolated from woody substrates, such as *Umbelopsis* representatives which are well-known late wood colonisers^29^. Therefore, rather than degrading complex substrates, they probably feed on substrates which were decomposed by other organisms^30^. Although there is currently no proteome data available for any Umbelopsidaceae representative, it is certain that for such a group of organisms membrane transporters would be of crucial value.

Schwartze *et al*.^20^ observed several expansions in genes coding of transporters in the *Lichtheimia corymbifera* genome. Our results are consistent with this finding, as the representatives of Lichtheimiaceae, Cunninghamellaceae and Syncephalastraceae were characterised by a higher number of protein-coding genes for membrane transporters and by more efficient growth on simple carbohydrates.

As expected, monosaccharides were generally metabolised faster than complex carbohydrates. The monosaccharide which was used the fastest was D-xylose, which is a pentose type sugar, first isolated from wood. All tested saprotrophic Mucoromycotina strains were also able to grow on xylan as a sole carbon source. These results reveal that xylan degradation capacity is much more widespread within Mucoromycotina representatives than previously thought^26^ and it indirectly proves their affinity for plant relations, set as a characteristic of this group by Spatafora *et al*.^7^.

Although Eichlerova *et al*.^31^ and Baldrian *et al*.^21^ showed that closely related fungal strains also reveal high similarity in enzyme production, they concluded that fungal ecophysiology is the main factor shaping fungal enzymatic capacities. In our study, the isolation substrate influence was best represented in D-Glucuronic acid usage which was metabolised more efficiently by strains isolated from dung. D-Glucuronic acid is a sugar acid derived from glucose, which is present in urine and therefore, the ability of dung-originating fungi to use it as a carbon source is not surprising.

As dung and soil are heterogeneous substrates, they are populated by both plant and animal related fungi. This diversity of organisms associated with organic debris is expected to show differences in carbon usage preferences (as can be seen on Fig. 3b). For example, although many fungi are known to be able to use uronic acids as a sole carbon source, the pathways for their catabolism are understudied. The first fungal metabolic pathway of uronic acids has been described only recently in *Aspergillus niger* (Ascomycota)^32^. The catabolic D-glucuronate pathway in this organism differs fundamentally from the pathways known from bacteria or animals. As uronic acids are typical for mucoralean cell walls, D-glucuronic acid usage and catabolic pathway responsible for degrading it is a particularly interesting in case of Mucoromycota representatives^33^. Moreover, the ability to degrade or modify them can influence the competitiveness of a particular fungus.

Although phylogenetic placement seems to be a reliable predictor of carbon assimilation capacities for Umbelopsidaceae, Lichtheimiaceae, and Cunninghamellaceae, this dependency cannot be seen for Mucoraceae. It can likely be explained by the heterogeneity of this family. *Mucor* is the largest and the most variable genus among the whole Mucoromycotina^34^. This variability is also reflected in the carbon assimilation capacities of its representatives and therefore makes it more difficult to draw any conclusions on this group’s traits’ similarity at the species, genus or family level.

The results of the study have revealed that neither phylogenetic distance nor strain isolation origin determines carbon assimilation capacities of saprotrophic Mucoromycotina representatives (i.e. Umbelopsidales and Mucorales). However, several significant differences in the growth capacity on specific carbon sources were observed between the representatives of different families. Most importantly, our results show that saprotrophic Mucoromycotina representatives are metabolically versatile and are able to use a wide variety of carbon sources including biopolymers of plant origin.

## Materials and Methods

### Isolates and culturing

72 strains belonging to 62 species of Mucorales and Umbelopsidales were provided by the Westerdijk Fungal Biodiversity Institute, Utrecht, Netherlands. Fungi were incubated for 10 days on different culture media (MEA, OA, CMA, PDA) and in different temperatures according to the recommendations received from the Westerdijk Fungal Biodiversity Institute culture collection. The culture conditions are indicated in Supplementary Table S1. The identity of all strains was confirmed by the sequencing of the internal transcribed spacer (ITS) region and with standard morphological identification procedures^35^.

### Determination of assimilation profiles for simple carbon substrates

Phenotypic microarray plates for filamentous fungi, FF (Biolog Inc., USA) were used to test the capacity of all 72 strains to grow on 95 different carbon sources (Supplementary Table S3) and one negative control – no carbon source added. Carbon sources were grouped into guilds according to Preston-Mafham *et al*.^36^. Spores were suspended in FF inoculation fluid with a deficient amount of carbon (Biolog Inc., USA) to produce a final optical density of 0.036 A at 590 nm. Spore suspensions were then inoculated on FF microplates and incubated in the aerobic Omnilog incubator plate reader (Biolog Inc., USA) for 96 h at 20 °C or 36 °C (according to optimal growth temperature recommended by Westerdijk Fungal Biodiversity Institute culture collection; Supplementary Table S1). The metabolic activity was measured kinetically by determining the colorimetric reduction of a tetrazolium dye approximately every 10 minutes over a 96 hour period. Colorimetric values for wells containing carbon substrates were blanked against the control well. Preliminary data analysis was done using the Biolog Kinetic and Parametric software (Biolog Inc., USA). The analysis of each strain was done in three replicates. The result was considered positive when a difference between the metabolic activities of the first and last day of incubation was observed in all three repetitions. The mean values and standard deviations of AUC (area under curve as described in Preston-Mafham *et al*.^36^) were calculated in order to evaluate the repeatability (Supplementary Table S2). 52 strains, representing 44 species belonging to Mucorales and Umbelopsidales, which passed the repeatability evaluation, were used for further carbon assimilation profiles analysis.

### Determination of growth capacity on selected biopolymers

36 strains of well sporulating cultures were tested for their capacity to grow on cellulose, xylan, starch and pectin as a sole carbon source. All samples were first cultured on MEA at 25 °C for one week and then they were transferred onto screening agar plates. The medium for all tests contained: 3 g of NH_4_NO_3_, 1 g of KH_2_PO_4_, 0.5 g of MgSO_4_ · 7H_2_O, 0.5 g of KCl, 15 g of agar, 1000 ml of water^22^. This medium was used as a negative control. For screening experiments, the medium was supplemented with 1% of cellulose powder, 0.5% of oat xylan powder, 1% of starch powder, or 10% of apple pectin respectively. Amylase activity was verified using 10% iodine to visualise the hydrolysis zone. All tests were done in three replicates.

### Data analysis

The average usage of each carbon source tested on Biolog FF microplates was represented as medians and ranges of AUC values in boxplot (Fig. 2). For 52 tested fungal strains, the metabolic capacity to grow on a particular substrate was represented as a heatmap of binary data (Fig. 1). The principal component analysis for all tested isolates was performed using *prcomp* function on Pearson correlation matrices with centring set to true. The phylogenetic placement (considered as family assignment according to Hoffmann *et al*.^10^) and strain isolation origin were used to explore factors shaping carbon usage capacities (Fig. 3). Finally, the significance of particular carbon utilisation capacity between different family representatives and between strains isolated from different substrates were calculated using the Kruskal–Wallis H test (also called one-way ANOVA on ranks) as the data did not fit a normal distribution pattern (Supplementary Table S3). Boxplots, representing the median AUC value of all fungi belonging to a particular group (ie. family or origin) growing on a particular carbon source, were drawn only for those substrates for which the difference in usage either by different families (only for families represented by more than one strain) or different trophic groups was statistically significant in the Kruskal-Wallis H test (p < 0.05) (Fig. 4). The correlation between phylogenetic distance and carbon assimilation dissimilarity matrices (Bray-Curtis) was calculated using the Mantel test (10000 permutations; Pearson correlation). All analyses were performed in R^37^ (including packages ggfortify v0.4.1^38^, vegan v2.4.2^39^ and ape v5.2^40^).

### Phylogenetic tree

To assess phylogenetic relationships between strains, we used publicly available nucleotide sequences of 18S rRNA gene, ITS fragment and 28S rRNA gene for each species used in the study (see Supplementary Table S4 for NCBI identifiers of sequences used). Alignments for each marker were done independently using the MAFFT v7 algorithm with -auto option^41^. The best-fitting evolution model was established for the alignment of each marker independently using modeltest-ng^42^. Alignments were then trimmed with trimal using -automated1 function and concatenated afterwards. For constructing the phylogenetic tree we used raxml-HPC v.8.2.4^43^, substitution model GTRGAMMAIX (as it was the best model for all three markers according to modeltest-ng), and 1000 bootstrap replicates.

### Transporter annotation

Few Mucorales strains have their genomes and predicted proteomes deposited in the NCBI genome database^44^. All 19 available Mucorales proteomes were downloaded in April 2018 from the aforementioned database and searched using pfam_scan.pl as a wrapper for HMMER with e-value threshold of 0.001 against the whole PFAM 31 database^45^. Pfam_scan.pl resolves overlapping hits and provides unambiguous protein domain architecture annotation. The transporter families mentioned by Schwartze *et al*.^20^ (MFS, ABC and other sugar transporters) were selected and compared between strains from different substrates and between different family representatives.

## Supplementary information

Supplementary information

Dataset 1

## Data Availability

All raw data generated for this study are available in Supplementary Table 1.
