# Supplementary material for: Carbon assimilation profiles of mucoralean fungi show their metabolic versatility
Source: Sci Rep. 2019 Aug 14;9:11864. doi: 10.1038/s41598-019-48296-w (PMC6694110; doi:10.1038/s41598-019-48296-w)
Supplement: Supplementary file 1 — Supplementary information [file 41598_2019_48296_MOESM1_ESM.docx]

**Carbon assimilation profiles of mucoralean fungi show their metabolic versatility**

Julia Pawłowska^1^, Alicja Okrasińska^1*^, Kamil Kisło^1^, Tamara Aleksandrzak-Piekarczyk^2^, Katarzyna Szatraj^2^, Somayeh Dolatabadi^3^, Anna Muszewska^2^

^1^ Department of Molecular Phylogenetics and Evolution, Faculty of Biology, Biological and Chemical Research Centre, University of Warsaw, Zwirki i Wigury 101, 02-089 Warsaw, Poland

^2^ Institute of Biochemistry and Biophysics, Polish Academy of Sciences, Pawinskiego 5A, 02-106 Warsaw, Poland

^3^ Faculty of Engineering, Sabzevar University of New Technology, Sabzevar, Iran

***Author for correspondence**: Alicja Okrasińska, alis.ok@biol.uw.edu.pl, +48 22 552 67 27, Department of Molecular Phylogenetics and Evolution, Faculty of Biology, Biological and Chemical Research Centre, University of Warsaw, Zwirki i Wigury 101, 02-089 Warsaw, Poland.

**Supplementary Table S1.** List of strains used in the study and the metadata associated with them.

**Supplementary Table S2.** Raw reads of carbon assimilation capacities obtained via Biolog for 75 initially tested strains in three repetitions.

**Supplementary Table S3.** Results of Wilcoxon tests. Significance of differences of mean values of specific carbon usage between delimited groups. Differences significant at p<0.05 are highlighted.

**Supplementary Table S4.** List of NCBI identifiers used for phylogenetic analysis.

**Supplementary Table S5.** Genome data and transporter counts.
